# Thin film hydration versus modified spraying technique to fabricate intranasal spanlastic nanovesicles for rasagiline mesylate brain delivery: Characterization, statistical optimization, and in vivo pharmacokinetic evaluation

**DOI:** 10.1007/s13346-022-01285-5

**Published:** 2022-12-30

**Authors:** Mohamed Mahmoud Ali, Raguia Aly Shoukri, Carol Yousry

**Affiliations:** Department of Pharmaceutics and Industrial Pharmacy, Faculty of Pharmacy, Cairo University, P.O. Box 11562, Cairo, Egypt

**Keywords:** Rasagiline mesylate, Spanlastics, Thin film hydration, Modified spraying technique, Intranasal, Brain targeting

## Abstract

Rasagiline mesylate (RM) is a monoamine oxidase inhibitor that is commonly used to alleviate the symptoms of Parkinson’s disease. However, it suffers from low oral bioavailability due to its extensive hepatic metabolism in addition to its hydrophilic nature which limits its ability to pass through the blood–brain barrier (BBB) and reach the central nervous system where it exerts its pharmacological effect. Thus, this study aims to form RM-loaded spanlastic vesicles for intranasal (IN) administration to overcome its hepatic metabolism and permit its direct delivery to the brain. RM-loaded spanlastics were prepared using thin film hydration (TFH) and modified spraying technique (MST). A 2^3^ factorial design was constructed to study and optimize the effects of the independent formulation variables, namely, Span type, Span: Brij 35 ratio, and sonication time on the vesicles᾽ characteristics in each preparation technique. The optimized system prepared using MST (MST 2) has shown higher desirability factor with smaller PS and higher EE%; thus, it was selected for further in vivo evaluation where it revealed that the extent of RM distribution from the intranasally administered spanlastics to the brain was comparable to that of the IV drug solution with significantly high brain-targeting efficiency (458.47%). These results suggest that the IN administration of the optimized RM-loaded spanlastics could be a promising, non-invasive alternative for the efficient delivery of RM to brain tissues to exert its pharmacological activities without being dissipated to other body organs which subsequently may result in higher pharmacological efficiency and better safety profile.

**Graphical Abstract:**

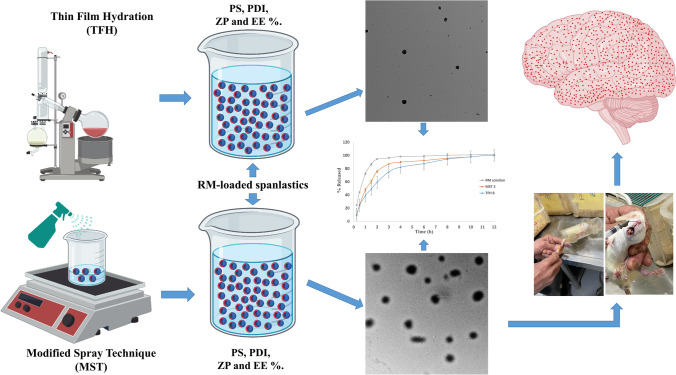

## Introduction

Parkinson’s disease (PD) is one of the most popular neurodegenerative diseases that develops after the loss of dopaminergic neurons in the substantia nigra and the accumulation of α-synclein, inside some cytoplasmic bodies called Lewy bodies [1]. The incidence of PD increases with aging, some environmental factors like exposure to carbon monoxide or pesticides in high levels, and other hereditary genetic factors. It is also more prevalent in males than females. Patients with PD show some motor symptoms like tremors, bradykinesia, and instability in the posture, in addition to non-motor changes such as sleep disorder, depression, hallucinations, dementia, and cognitive impairment. The diagnosis of the disease is based on the clinical symptoms and family history with no definite laboratory investigation except for histological observation of α-synclein inside Lewy bodies. Positron emission tomography and single photon emission computed tomography tests have been performed recently using dopaminergic ligand to indicate dopamine deficiency and metabolism which improved the certainty of the diagnosis [2]. The management and treatment of PD are usually directed towards the improvement of the clinical symptoms and prevention of more neuronal degeneration [3]. The treatment strategy always begins with restoring dopamine levels in the substantia nigra through the administration of levodopa (L-DOPA). As a common adjuvant with L-DOPA, monoamine oxidase type B (MAO-B) inhibitors such as rasagiline and selegiline are widely used. MAO-B inhibitors act to inhibit the degradation of dopamine by monoamine oxidase enzyme which prevents further decrease in the dopamine levels [4].

Rasagiline mesylate (RM) is a selective potent irreversible inhibitor of MAO-B that is used to restore dopamine levels inside the central nervous system (CNS). It is rapidly absorbed after oral administration to reach its maximum concentration in plasma within 1 h; however, it has a short half-life of 1.5–3.5 h [5]. RM shows low oral bioavailability as it is extensively metabolized by cytochrome p450. In addition, the hydrophilic nature and high water solubility of RM limit its ability to pass through the blood–brain barrier (BBB) and reach its active site [6].

Recently, the intranasal (IN) route for drug administration has become far superior to other conventional routes in improving the delivery of drugs into the central nervous system (CNS) through BBB [7]. The intranasally administered drug may reach the brain directly via the olfactory pathway or after absorption into the lymphatic system and then to the cerebrovasculature to reach the brain [8]. The direct movement through the olfactory pathway involves transcellular and paracellular penetration [9]. The intranasally administered drug can travel along the olfactory sensory nerves to reach olfactory bulb. After which, it can diffuse through the adjacent nerves present in the cerebrum to the different brain regions [10]. Thus, the IN route tends to overcome the first pass effect for the drugs that are extensively metabolized after oral administration. Subsequently, this gives an opportunity for lower doses and less side effects [11]. In addition, the large surface area of the nasal mucosa and the underlying rich vasculature provide high absorption rates and faster onset of action [12]. It can also be used for the delivery of large peptide macromolecules such as protein-based antigens [13]. Thus, the intranasal route was used for the novel treatment of CNS disorders like Parkinson’s disease and Alzheimer’s disease by stem cells [14]. Several commercial intranasal products are now available to treat CNS disorders like Nayzilam^®^ nasal spray developed by UCB, Inc., Belgium, and Nayzilam^®^, a midazolam spray, which was developed to stop acute seizure episodes [15]. Also, Zomig^®^ nasal spray (2.5 or 5 mg of zolmitriptan) was developed by AstraZeneca, Inc., Canada, to relieve acute migraine attacks [16, 17].

On the other hand, the pharmaceutical applications of nanoparticles have showed significant progress in the diagnosis and treatment of different disorders. The potential to manipulate the particle size, the surface charge, and the shape of the nanoparticles has contributed to their diverse wide applications as drug carriers and imaging agents [18]. Among their numerous advantages, their ability to pass through BBB after intranasal administration is considered one of the gold standard applications of nanoparticles development to study the pathogenesis and alleviate the symptoms of many CNS disorders. They can also be used for improving the therapeutic performance and preventing some of the peripheral and systemic side effects of some chemotherapeutic agents used for brain cancers [19]. Nanoparticles are also able to overcome the rapid intranasal mucocilliary clearance due to their rapid absorption aided by their small particle size and elasticity that permit the direct passage of the intranasally administered nanoparticles through the olfactory pathway to brain [20]. Spanlastics are flexible, biodegradable, and deformable nanovesicles that are composed mainly of Span as a non-ionic surfactant and edge activator (EA). They are characterized by their spherical unilamellar or multilamellar structure with an inner core that is able to encapsulate hydrophilic or lipophilic drugs and improve their bioavailability, thus protecting the encapsulated drug against the external environment and the extensive hepatic metabolism. The proper choice of the EA that provides the required degree of elasticity and deformability is a keypoint during spanlastic formulation to facilitate their passage through the tight junctions of the relatively impermeable membranes [21, 22]. In spite of their unique surface characters, few studies have investigated their potential application for IN administration of centrally acting drugs. Zolmitriptan-spanlastics were formulated for the treatment of migraine episodes where El-Nabarawy et al. [23] have found that the intranasally administered nanovesicles showed better alleviation of migraine episodes when compared to the in vivo outcomes of the available commercial products. IN spanlastics were also used to improve the brain-targeting properties of carbamazepine [24] and granisetron hydrochloride through its formulation in a nasal spanlastic gel insert [25].

The work in this study aims to formulate a new vesicular nanosystem to enhance the distribution of RM to the brain rather than the systemic circulation. To achieve our goal, RM-loaded spanlastic vesicular systems were prepared using different preparation techniques, namely, thin film hydration (TFH) and modified spraying technique (MST), to fabricate vesicles with small vesicular size and high drug encapsulation. 2^3^ full-factorial designs were constructed for both techniques using the same independent formulation variables, and the results of the formulated runs were statistically characterized relative to the values of their particle size (PS), polydispersity index (PDI), zeta potential (ZP), and entrapment efficiency (EE%), compared and optimized to generate the best compromise of all the selected formulation variables. The in vitro release profiles of RM from the optimized systems prepared by each technique were evaluated, and their morphological characteristics were investigated via transmission electron microscope (TEM). The pH of the optimized systems was also measured to ensure its safe intranasal administration. And finally, the optimized system with the smallest PS and highest desirability factor was selected for further in vivo pharmacokinetic evaluation to demonstrate its brain-targeting efficiency and its estimated in vivo performance.

## Methods

### Materials and methods

Rasagiline mesylate (RM), Span^®^60 (sorbitan monostearate), Span^®^80 (sorbitan monooleate), Brij 35 (polyoxyl 23 lauryl ether), and dialysis cellulose membrane (D9777-100FT, flat width 25 mm, 1400 molecular weight cut-off) were obtained from Sigma-Aldrich Inc. AL. (St. Louis, MO, USA). Sucrose, potassium chloride, sodium chloride, disodium hydrogen phosphate, hydrogen disodium phosphate, calcium chloride, and ethanol were purchased from EL-Nasr ln pharmaceutical chemicals company (Cairo, Egypt).

### Study design

RM-loaded spanlastic nanovesicles were prepared using two different preparation methods, namely, thin film hydration (TFH) technique and modified spraying technique (MST). A two-level, three-factor design (2^3^) was developed using Design-Expert 12^®^ software (Stat-ease Inc., Minneapolis, MN, USA) to study the effects of the different formulation variables on the characteristics of the prepared spanlastic nanovesicles in each preparation method. The factorial design is an experimental building methodology that is commonly used for studying the effects of the independent factors and their interactions on one or more selected variables [26]. The independent formulation variables investigated are Span type (Span^®^80 or Span^®^60; *X*_1_), Span: Brij 35 ratio (1:1 or 4:1; *X*_2_), and the sonication time (0 or 60 s; *X*_3_), whereas the formulated vesicles are characterized in terms of: particle size (PS; *Y*_1_), polydispersity index (PDI; *Y*_2_), zeta potential (ZP; *Y*_3_), and entrapment efficiency (EE%; *Y*_4_) as shown in Table 1. Each design is constructed of 8 runs in duplicates as shown in Tables 2 and 3. The statistical influence of the formulation variables on the nanovesicular properties was evaluated via analysis of variance (ANOVA) using Design-Expert 12^®^ where the statistical significance was considered at *p* value < 0.05.

**Table 1:** Levels of the independent formulation variables for a 2^3^ factorial design and the optimization criteria for the responses

Independent variables	Levels	
*X*_1_: Span type	Span^®^80	Span^®^60
*X*_2_: Span: Brij 35 ratio	1:1	4:1
*X*_3_: Sonication time (sec)	0	60

Responses	Optimization goal	
*Y*_1_: Particle size (nm)	Minimize	
*Y*_2_: Polydispersity index	Minimize	
*Y*_3_: Zeta potential (mv)	Maximize (absolute values)	
*Y*_4_: Entrapment efficiency (%)	Maximize

**Table 2:** Composition of RM-loaded spanlastics prepared via thin film hydration technique corresponding to the developed 2^3^ factorial design with their resultant dependent responses

System run	*X*_1_: Span type	*X*_2_: Span: Brij 35 ratio	*X*_3_: Sonication time (second)	*Y*_1_: PS ± SD (nm)	*Y*_2_: PDI ± SD	*Y*_3_: ZP ± SD (mV)	*Y*_4_: EE ± SD (%)
TFH 1	Span^®^60	1:1	0	164.631 ± 03.6	0.369 ± 0.007	(-)41.7 ± 1.58	47.75 ± 0.36
TFH 2	Span^®^60	1:1	60	211.766 ± 51.3	0.755 ± 0.088	(-)45.6 ± 0.10	56.46 ± 3.06
TFH 3	Span^®^60	4:1	0	537.065 ± 29.2	0.707 ± 0.055	(-)46.2 ± 2.55	58.21 ± 0.09
TFH 4	Span^®^60	4:1	60	232.916 ± 16.9	0.483 ± 0.066	(-)42.7 ± 0.70	50.11 ± 2.84
TFH 5	Span^®^80	1:1	0	251.233 ± 30.7	0.487 ± 0.028	(-)40.8 ± 2.05	52.30 ± 2.60
TFH 6	Span^®^80	1:1	60	172.816 ± 14.1	0.642 ± 0.037	(-)40.4 ± 0.86	48.71 ± 1.50
TFH 7	Span^®^80	4:1	0	250.666 ± 46.6	0.440 ± 0.027	(-)48.4 ± 1.23	45.09 ± 3.62
TFH 8	Span^®^80	4:1	60	151.333 ± 04.5	0.299 ± 0.037	(-)44.6 ± 2.03	50.33 ± 1.65

*PS* particle size, *PDI* polydispersity index, *ZP* zeta potential, *EE%* entrapment efficiency percentage

**Table 3:** Composition of RM-loaded spanlastics prepared via modified spraying technique corresponding to the developed 2^3^ factorial design with their resultant dependent responses

System run	*X*_1_: Span type	*X*_2_: Span: Brij 35 ratio	*X*_3_: Sonication time (second)	*Y*_1_: PS ± SD (nm)	*Y*_2_: PDI ± SD	*Y*_3_: ZP ± SD (mV)	*Y*_4_: EE ± SD (%)
MST 1	Span^®^60	1:1	0	103.509 ± 6.2	0.333 ± 0.038	(-)32.8 ± 1.66	56.17 ± 0.03
MST 2	Span^®^60	1:1	60	84.060 ± 4.5	0.654 ± 0.097	(-)38.3 ± 2.30	53.60 ± 1.90
MST 3	Span^®^60	4:1	0	522.666 ± 34.0	0.703 ± 0.033	(-)33.1 ± 1.13	46.29 ± 2.36
MST 4	Span^®^60	4:1	60	359.183 ± 33.9	0.540 ± 0.069	(-)30.1 ± 2.10	51.01 ± 3.05
MST 5	Span^®^80	1:1	0	143.266 ± 9.2	0.409 ± 0.013	(-)37.9 ± 0.23	38.42 ± 3.15
MST 6	Span^®^80	1:1	60	165.280 ± 48.3	0.583 ± 0.059	(-)39.6 ± 0.85	42.41 ± 0.99
MST 7	Span^®^80	4:1	0	266.750 ± 30.1	0.496 ± 0.016	(-)43.6 ± 4.45	38.03 ± 0.62
MST 8	Span^®^80	4:1	60	163.083 ± 23.1	0.405 ± 0.110	(-)39.4 ± 1.73	43.49 ± 5.70

*PS* particle size, *PDI* polydispersity index, *ZP* zeta potential, *EE%* entrapment efficiency percentage

### Preparation of RM-spanlastic vesicles

#### Thin film hydration technique

RM-loaded spanlastics are prepared by TFH [27] as per the formulation variables shown in Table 2. Briefly, Span^®^60 or Span^®^80 was dissolved in 2 mL ethanol in a round-bottom flask which was then evaporated under vacuum using rotary evaporator (Rotavapor, Heidolph, 1300 w, Schwabach, Germany) at 60 °C and 90 rpm. A thin dried film was formed which was hydrated by 5 mL phosphate-buffered saline (PBS) containing RM (20 mg) and Brij 35 at 60 °C and 150 rpm to formulate the spanlastic dispersion. The prepared dispersion was then sonicated in a water bath sonicator (Elma, Elma Schmidbauer GmbH, Singen, Germany) to reduce the particle size [28]. Finally, the obtained spanlastic vesicular systems were subjected to four consecutive freeze–thaw cycles at –8 °C for 8 h and 25 °C for 1 h aiming to enhance the entrapment of RM inside the nanosystem [29].

#### Modified spraying technique

RM-loaded spanlastic vesicles are also prepared using MST [30, 31] as per Table 3. Concisely, Span^®^60 or Span^®^80 (100 mg), Brij 35, and RM (20 mg) were dissolved in 2 mL ethanol to form the organic phase which was then transferred to a spray device. The aqueous phase was prepared of sucrose solution (9% w/v in double-distilled water) and heated to 60 °C in a closed system. The organic phase was then sprayed on the aqueous medium at a rate of 250 µL each 5 s while stirring at 1500 rpm and 60 °C (230 V-50/60 HZ, DAIHAN Scientific Co., Ltd. South Korea). Finally, the formulated nanodispersions were subjected to freeze–thaw cycles as previously described. All systems were prepared randomly in duplicates.

### Characterization of RM-loaded spanlastics

#### Determination of particle size, polydispersity index, and zeta potential

The PS, PDI, and ZP values for all systems were measured by photon correlation spectroscopy using Malvern Zetasizer (Malvern Panalytical Ltd., Malvern, UK). Briefly, all samples were diluted with double-distilled water [32] and measured at 25 °C. The mean individual values for two replicate batches (each with three measurements) are determined, and their mean values ± SD are presented in Tables 2 and 3.

#### Determination of RM entrapment efficiency (EE%)

The entrapment of RM inside the spanlastic vesicles was determined after the separation of the unentrapped drug using dialysis method [33]. In brief, 2 mL of each system was enclosed in a dialysis bag and immersed in 25 mL of double-distilled water for 4 h, which was previously estimated to ensure the complete liberation of free unentrapped drug to the outer compartment. The amount of RM in the outer compartment was then determined spectrophotometrically at λ_max_ 265 nm (Shimadzu UV-1800, kyoto, Japan) [34]. Finally, the EE% of RM was calculated using the following Eq. (1) [35]:

1 $$\mathrm{Entrapment\ efficiency}\ \%\mathrm{\left(EE\%\right)}=\frac{( Total\ drug\ amount-free\ unentrapped\ drug)}{drug\ amount } \times 100$$

The individual values for two replicates were determined and their mean values were reported.

### Optimization of RM-loaded spanlastic

The different independent variables for the formulation of RM-loaded spanlastics were optimized using Design-Expert 12^®^ software. The optimization criteria are set to minimize the PS and PDI while maximizing the absolute ZP values and EE% as shown in Table 1. The same optimization criteria were applied to both designs. The optimized systems obtained from both techniques were prepared and used for further evaluation.

### Characterization of the optimized systems

#### In vitro release characteristics of RM

The in vitro release of RM from the optimized systems was investigated using modified Franz diffusion cells (Franz diffusion cell with dialysis membrane) [36]. In a few words, an exact volume of each optimized system was placed in the donor cell, and the dialysis process was performed against 50 mL of simulated nasal fluid (SNF; CaCl_2_.2H_2_O 0.32 mg/mL, NaCl 7.45 mg/mL, KCl 1.29 mg/mL, pH 6.5) [37] maintained at 37 °C while stirring at 50 rpm [38]. Three milliliter sample was withdrawn at each time interval (0.25, 0.5, 1, 1.5, 2, 3, 4, 6, 8, 10 and 12 h) and replaced with equal volumes of drug free SNF [39]. The amount of RM in each sample was measured spectrophotometrically at λ_max_ 265 nm. The in vitro release profiles of the two optimized systems were compared to RM aqueous solution profile. Furthermore, the mean dissolution time (MDT) was calculated for both systems following Eq. (2) [40]:

2 $$MDT=\frac{\sum_{j=1}^{n}{t}_{j}^{*}{\Delta M}_{j}}{\sum_{j=1}^{n}{\Delta M}_{j}}$$

where *j* is the sample number, *t*_j_* is the midpoint time between *t* and *t*_*j*-1_ that can be calculated by (*t* + *t*_*j*-1_/2), *n* is the number of release samples, and ∆*Mj* is the additional amount released between *t* and *t*_*j*-1_. The higher the MDT value, the slower the drug release rate.

#### Transmission electron microscopy (TEM)

The morphological characteristics of the two optimized systems were evaluated by transmission electron microscope (Joel 1400, Tokyo, Japan). The micrographs were prepared by diluting one drop of spanlastic dispersion followed by adsorption on carbon-coated copper grids [41]. The excess dispersion was removed with a filter paper, and then, the sample was left to dry at room temperature for 10 min prior to microscopic visualization at 80 kv [42].

#### pH measurement

The pH of the optimized systems was measured to ensure their safety upon application into the nasal mucosa. Briefly, a definite volume of each optimized system was diluted with double distilled water in ratio (1:10), and then, the pH was measured using a calibrated pH meter (Jenway 3510, Jenway, Staffordshire, UK) [43].

### In vivo evaluation of RM-loaded spanlastics

In order to evaluate the in vivo performance of the selected MST 2, an in vivo pharmacokinetics study was performed to measure the bioavailability and brain-targeting ability of the selected system after intranasal administration into rats. The study protocol was previously approved by the research ethics committee for the experimental and clinical studies at the Faculty of Pharmacy Cairo University with approval code (PI 2680). Briefly, forty-eight rats were divided into two groups (*n* = 24), and each group was divided into eight subgroups (*n* = 3). The average weight of the rats was 180 gm, and they were subjected to 12-h cycles of light/darkness. They were kept in cages with free access to food and water. The relative humidity was maintained in range 40–60%, and the temperature ranged from 20 to 25 °C during the experiment.

The first group was administered the optimized MST 2 equivalent to 0.05 mg/kg/day of RM [44] intranasally (IN) using a polyethylene tube which was attached to Hamilton syringe, whereas the second group was administered the same dose of an intravenous (IV) RM aqueous solution through the tail vein. Blood samples were collected from the rats at specified time intervals (0.25, 0.5, 1, 1.5, 2, 4, 8, and 12 h) via the puncture of lateral vesicular vein, centrifuged at 4000 rpm for 15 min, and separated to obtain the plasma that was then frozen at –80 °C until further investigations. Additionally, six rats (three of each subgroup) were anaesthetized and sacrificed to separate the brain tissues at 0.5, 1, 2, 4, and 8 h. The brain tissues were washed twice with normal saline and then weighed. An equal weight of normal saline was added to each brain sample, which was then homogenized (Heidolph DIAX 900, Heidolph Instruments, Schwabach, Germany) and kept frozen at – 80 °C until analysis. RM levels in plasma samples and brain homogenates were measured by a validated liquid chromatography-mass spectrometry (LC–MS/MS) method.

#### Sample preparation

Calibration curves of RM in plasma and brain homogenate were constructed using clonazepam as internal standard (IS). Briefly, specific volumes of RM stock solution were mixed with one hundred microliter of clonazepam stock solution (100 ng/mL) and then spiked into 0.5 mL plasma or brain homogenate to obtain the following concentrations: 0.1, 1, 10, 20, 100, and 1000 ng/mL. For RM extraction from the samples, 0.5 mL of plasma samples or brain homogenates was mixed with 100 µL of clonazepam solution as IS and 4 mL of ethyl acetate to precipitate the proteins followed by vortexing (Stuart SA8, BiBBY Sterlin Ltd., UK) for 5 min and centrifugation for 10 min at 3000 rpm. The supernatant was transferred to a new tube to evaporate the organic solvent under vacuum using a vacuum concentrator (Eppendorf 5301, Hamburg, Germany). The obtained dry residue was reconstituted in 0.5 mL of the mobile phase (acetonitrile: 0.1% formic acid solution in water at a ratio (80:20 v/v)) after then, 9 µL of the obtained solution was injected into (LC–MS/MS) for analysis.

##### LC–MS/MS

The analysis of RM in plasma samples and brain homogenates was done by LC–MS/MS (Shimadzu CBM20A, Japan) fitted with mass spectrometer AB Sciex 4000 (Sciex Instruments, USA) and auto-sampler (Shimadzu SIL20A, Japan). The mass spectrometer was equipped with turbo ion spray having a positive polarity with temperature set at 500 °C. The samples were analyzed by injecting aliquots of 9 µL, and the mobile phase flow rate was adjusted at 0.8 mL/min. The transition was from 172.036 to 117.000 m/z for RM and from 316.024 to 270 m/z in case of clonazepam. The analysis of the obtained results was done using Analyst software (version 1.6.3, Sciex Instruments, Framingham, USA).

#### Pharmacokinetic study

Non-compartmental pharmacokinetic model was applied to analyze RM plasma and brain levels using Kinetica software (version 2000, Arlington, USA). There are different pharmacokinetic parameters, namely, *C*_max_ (maximum concentration in brain and plasma), *T*_max_ (time to reach maximum concentration), AUC_0-∞_ (area under the curve), and AUMC_0-∞_ (area under first moment curve). AUC and AUMC were calculated by the trapezoidal rule; MRT (mean residence time; MRT = AUMC_0-∞_/AUC_0-∞_) [45], half-life, and elimination rate constant (*k*) were calculated for the intranasally administered MST 2 and the intravenous RM solution. In order to ensure the brain-targeting ability of the formulated system, the brain-targeting efficiency (BTE%) which compares the specific delivery of drug to brain after IN administration and IV administration was calculated as per Eq. (3) [46]:

3 $$BTE\ \%= \frac{^{{B}_{IN}}/_{{P}_{IN}}}{^{{B}_{IV}}/_{{P}_{IV}}} \times 100$$

where *P*_*IN*_ and *B*_*IN*_ are the AUC_0-∞_ in the plasma (*P*) and brain (*B*) after intranasal administration of the optimized system, while P_IV_ and B_IV_ are the AUC_0-∞_ after IV administration. BTE% values above 100 indicate efficient delivery of the drug to the brain after IN administration [47].

In addition, direct transfer percentage (DTP%) was also calculated to measure the extent of RM reaching the brain directly from the nose relative to the total amount reaching the brain after IN administration. DTP% was calculated following Eqs. (4) and (5) [48]:

4 $$DTP \%= \frac{\left({B}_{IN }-{B}_{x}\right)}{{B}_{IN}} \times 100$$

5 $${B}_{x}= \frac{{B}_{IV}}{{P}_{IV}} \times {P}_{IN}$$

where *B*_*x*_ is RM fraction that reached brain through BBB from the systemic circulation after intranasal administration. Positive values for DTP% suggest that the drug passes directly from the nose to the brain after IN administration, while zero or negative values indicate that the drug reaches the brain after being cleared from the nasal cavity to blood stream and then pass through BBB [49]. The pharmacokinetic parameters were analyzed statistically by ANOVA at *p* value < 0.05 using SPSS (SPSS^®^ statistics program software, IBM, USA).

## Results and discussion

The blood–brain barrier (BBB) is a term used to describe the non-fenestrated nature of the microvasculature reaching the CNS. Its tight junctions hinder the movement of ions, molecules, cells, and most therapeutic compounds across their wall to the brain tissues [50]. The PS of the different compounds plays a crucial role in enhancing its transportability across the BBB where the endocytosis of different components can be enhanced by reducing their PS and increasing their surface area [51]. It was previously claimed that nanoparticles less than 100 nm show the highest potential for delivering most drugs through BBB [52]. In this study, we managed to prepare RM-loaded spanlastics using 2 different techniques, TFH and MST, aiming to produce nanovesicles with small PS and high EE%. TFH is a commonly used technique for the preparation of nanovesicles because of its ability in producing a uniform nanodispersion [53]. In our preliminary trials, TFH was successfully adopted to formulate RM-loaded spanlastics with high EE%; however, it was incapable of producing vesicles with PS less than 100 nm; thus, MST was applied to produce vesicles with smaller PS. The preparation of nanovesicles via the novel spraying methods is simple and results in vesicles with small PS which may enhance their activity when compared to the larger particles prepared by the conventional methods [54]. Spraying techniques are considered “break-down” methods for the conversion of a liquid solution into nanoparticles. The characteristics of the final nanoparticles such as PS and surface morphology can be modified by adjusting the composition and the concentration of the starting solution as well as the size of the atomized droplets [55]. The modified spraying technique (MST) is considered a modification of the ethanol injection method to produce more uniform and size-controlled nanovesicles with high surface area [30]. Our preliminary results suggested that MST is more efficient in reducing PS than the conventional ethanol injection method which may be explained by the difference in the organic phase injection method in both techniques where the ethanol injection depends on the rapid injection of the organic phase in a continuous stream [56], whereas MST works via spraying small organic phase droplets in the aqueous medium. It is worth mentioning that several atomizers were tested in the screening stage and the one resulted in the smallest PS of RM-loaded spanlastics was selected. The use of ethanol in both preparation techniques was attributed to its beneficial impact on producing smaller vesicles, increasing ZP and EE%. Ethanol can impart a certain degree of steric stabilization to the formulated vesicles owing to its ability in interpenetrating the lipid bilayers which results in smaller vesicular size [57]. Additionally, ethanol has a condensing ability on the lipid membranes as it causes breakdown of large unilamellar vesicles (LUVS) to small multilayer vesicles which enhances RM partitioning inside the lipid bilayer [58, 59]. Furthermore, the penetrability of ethanol through the vesicles layers provokes the expulsion of the counter ions out of the vesicles which imparts more negative charge to the membrane and subsequently higher ZP values [60].

### Statistical analysis

RM-loaded spanlastic nanovesicles were prepared by TFH and MST in which 2^3^ factorial design was constructed to study the effects of the different formulation variables on the characteristics of the formulated RM-spanlastic nanovesicles using both techniques. Design-Expert 12^®^ software was used to analyze the variables affecting the dependent responses. The three-factor interaction model (3FI) was suggested by the software to evaluate all the dependent responses in both techniques except for the EE% in MST which was analyzed by the main effect model. The selected model orders showed the highest *R*^2^ values, high adjusted and predicted *R*^2^ values with the least difference between them, the lowest predicted residual error sum of squares (PRESS) values, and adequate precision values higher than 4 suggesting the high discriminating performance of the built models [61]. The predicted *R*^2^ values of the models are used to measure the predictability of the built designs. As previously stated in the literature [62], the adjusted *R*^2^ and predicted *R*^2^ are in good correlation when the difference between their values does not exceed 0.2. The statistical ANOVA values for the different responses in both preparation techniques are shown in Table 4. All *p* values were < 0.05, which indicates the statistical significance of the built models.

**Table 4:** Summary of model statistics for the measured responses of RM-loaded spanlastics prepared by TFH and MST methods

	TFH				MST			
Response	PS (nm)	PDI	ZP (mV)	EE (%)	PS (nm)	PDI	ZP (mV)	EE (%)
Model	3FI	3FI	3FI	3FI	3FI	3FI	3FI	Main effect
*R*^2^	0.9307	0.8724	0.6509	0.6442	0.9537	0.6652	0.7636	0.7309
Adjusted *R*^2^	0.8845	0.8087	0.5637	0.5895	0.9305	0.5815	0.7045	0.6636
Predicted *R*^2^	0.7809	0.6735	0.3794	0.4611	0.8814	0.4048	0.5797	0.5216
*p *value	< 0.0001	0.0003	0.0044	0.0012	< 0.0001	0.0035	0.0005	0.0010
Adeq. precision	13.9057	10.1994	8.1371	7.7240	17.4029	7.8518	10.2505	8.1520
PRESS value	50,027.29	0.1340	95.050	191.76	38,740.08	0.1784	148.730	371.97

*TFH* thin film hydration, *MST* modified spraying technique, *PS* particle size, *PDI* polydispersity index, *ZP* zeta potential, *EE%* entrapment efficiency percentage, *3FI* three factorial interaction, *adeq.precision* adequate precision, *PRESS* predicted residual error of sum squares

### Evaluation of RM spanlastic vesicles

#### Effect of the independent variables on the particle size

**Fig. 1:**
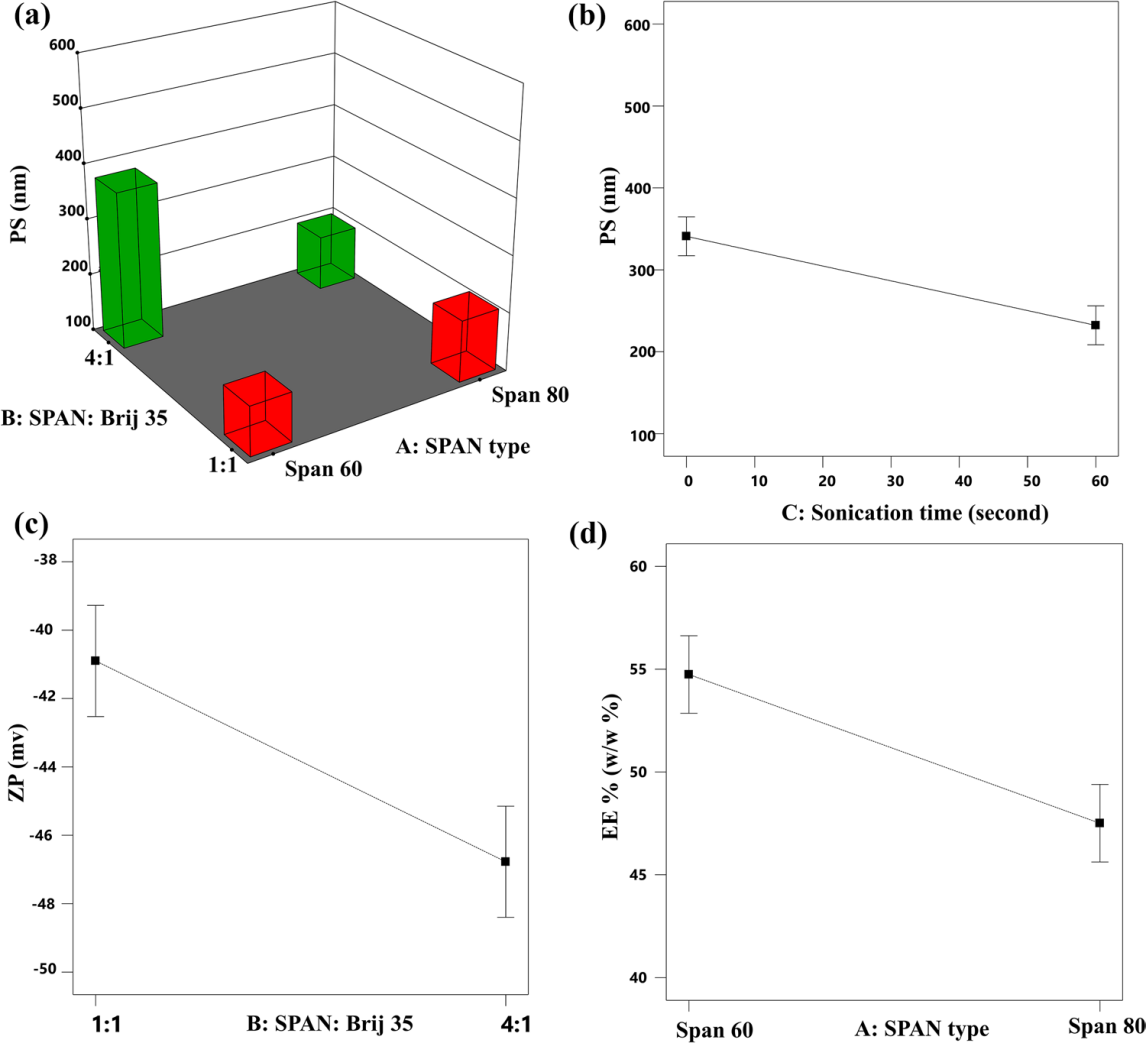
3D surface plot for the effect of Span type and Span: Brij 35 ratio on the PS (**a**), line plot for the main effect of sonication time on the PS (**b**), the main effect of Span: Brij 35 ratio on the ZP (**c**), and the main effect of Span type on the EE% (**d**) of RM-loaded spanlastics prepared by TFH

**Fig. 2:**
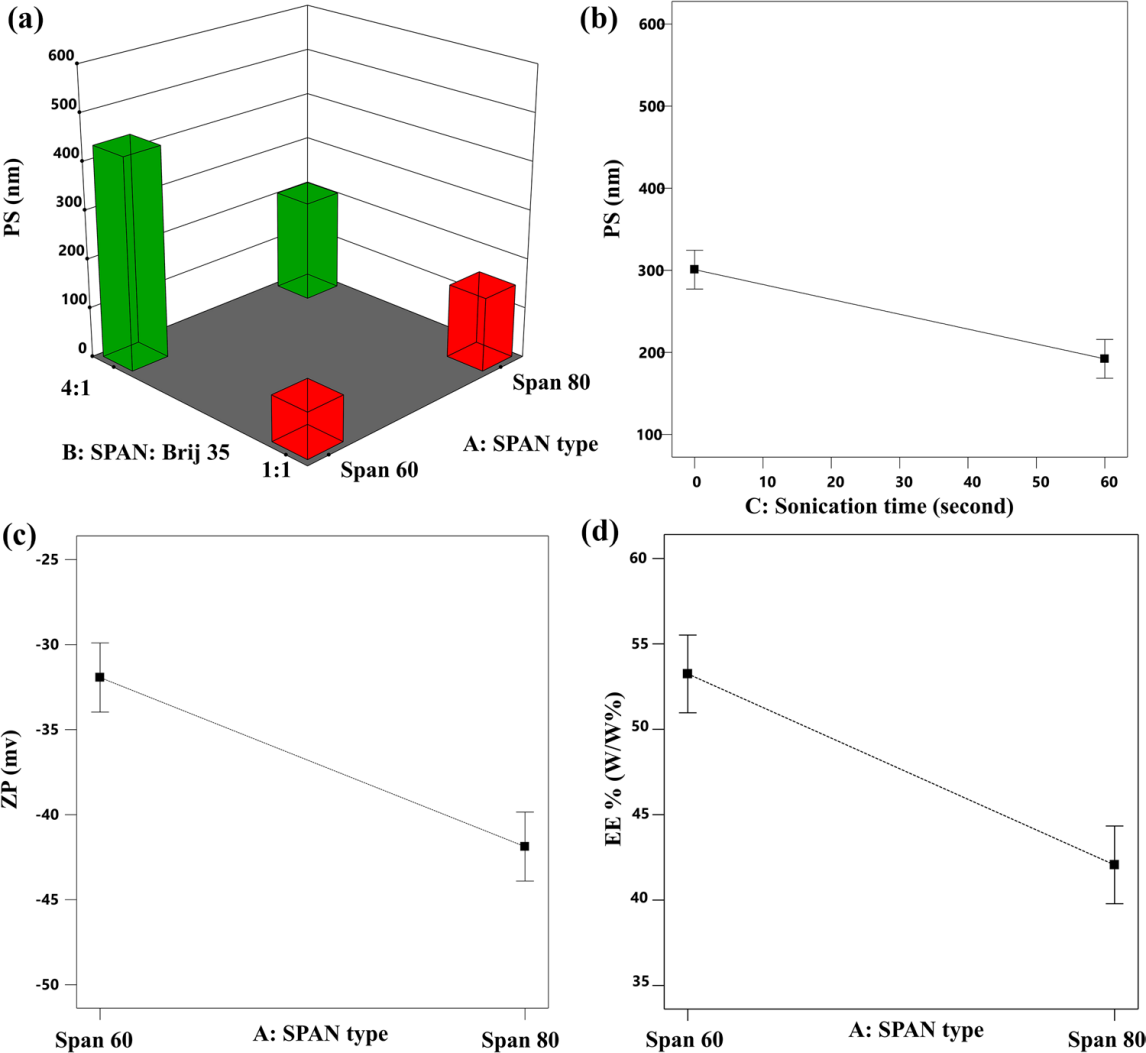
3D surface plot for the effect of Span type and Span: Brij 35 ratio on the PS (**a**), line plot for the main effect of sonication time on the PS (**b**), the main effect of Span type on the ZP (**c**), and EE% (**d**) of RM-loaded spanlastics prepared by MST

The PS of the formulated nanovesicles is an important parameter that affects the direct transfer of the vesicles from the nose to the brain as it was previously reported [63] that nanoparticles with size smaller than 100 nm may pass directly to the brain via olfactory pathway. In our study, the PS of the vesicles obtained with TFH was from 151.333 to 537.065 nm, whereas the PS of the formulated nanovesicles ranged from 84.060 to 522.666 nm in MST. These results support the previous claim that smaller nanovesicles can be easily formulated using spraying techniques. Statistical analysis of the data in both techniques revealed that the Span type, Span: Brij 35, and sonication time significantly affected the vesicles’ size. ANOVA results have shown that the PS of the formulated spanlastics was significantly affected by the type of Span used with *p* values of 0.0041 and 0.0017 for TFH and MST, respectively. The effect of each Span type differs according to the amount of EA used as there was a significant interaction between the type of Span and Span: Brij 35 ratio with respective *p* values 0.0008 and < 0.0001 for TFH and MST. Meanwhile, the effect of Span: Brij 35 ratio on the spanlastics᾽ size was also significant with *p* = 0.0016 for TFH and < 0.0001 for MST. In general, increasing the amount of EA to a ratio of 1:1 results in vesicles with lower PS as shown in Figs. 1a and 2a. This reduction in the PS values could be correlated with the lower interfacial tension observed with higher amounts of the EA [24, 64]. In addition, the lower amounts of EA used at the 4:1 ratio could be insufficient to surround the whole surface of the formulated vesicles which may result in a hindered emulsification process and subsequent particles coalescence to produce larger vesicles with smaller active surface area that can be efficiently stabilized with the available EA molecules [65]. These results could also be explained based on the structure of Brij 35 in water. Low concentrations of Brij 35 may result in a more diluted solution with higher chance for the formation of extended and less twisted hydrocarbon chains which may lead to the formation of vesicles with larger sizes [66]. This effect of Span: Brij ratio on the PS of the formulated spanlastics was more significant when Span^®^60 was used. This could be attributed to the difference in the structure between Span^®^60 and Span^®^80. Span^®^80 is characterized by the presence of double bond in the eighth carbon [67] which gives their molecules flexible structure and ability to bend their chain in the spanlastic layers, while other types of Span such as Span^®^60 and Span^®^20 possess saturated hydrocarbon chains with no double bond; thus, they have lower tendency to be accommodated effectively inside the vesicle layers [68] especially in the presence of low amounts of EA (ratio 4:1) resulting in larger PS.

Finally, it is observed that the nanovesicular dispersions subjected to longer time of sonication during preparation resulted in spanlastics with smaller PS either with TFH (*p* = 0.0006) or MST (*p* = 0.0068) as illustrated in Figs. 1b and 2b, respectively. This effect could be attributed mainly to the prolonged impact of the ultrasonic waves on the formulated vesicles. These waves tend to form cavitation bubbles within the spanlastics’ layers, which then blow and shatter the large spanlastic vesicles into more smaller vesicles [69].

#### Effect of independent variables on the zeta potential

Zeta potential is an important parameter to evaluate the physical stability of the colloidal dispersions. Nanoparticle dispersions with ZP values more than 30 (absolute values) are considered sufficiently stable and not liable to aggregation [70].

In our study, the ZP values of the spanlastic vesicles formulated using TFH were in range of − 48.43 to − 40.4 mV, while those prepared by MST ranged from − 43.6 to − 30.1 mV, suggesting that the formulated vesicle are highly stable devoid from any aggregated particles [71]. The statistical analysis of data shows that the ZP of the vesicles formulated using TFH was significantly affected (*p* = 0.0083) by the Span: Brij 35 ratio as shown in Fig. 1c where higher ZP values were observed at higher Span: Brij 35 ratio (4:1). This can be explained based on the effect of EA concentration on the PS. The higher vesicles’ size attained at 4:1 Span: Brij 35 ratio may reduce the electrical mobility of the vesicles and increase their ZP values [72]. In contrast, the ZP values in MST are significantly (*p* = 0.0003) affected by the type of Span applied during their preparation as shown in Fig. 2c. This can also be explained in the light of the structural difference between Span®80 and Span®60 where Span®80 is characterized by the presence of unsaturation in the eighth carbon. It was previously reported by Park et al. [73] that the higher the degree of unsaturation in the vesicular components, the higher the ZP values.

#### Effect of independent variables on the entrapment efficiency

The entrapment of RM inside the spanlastic vesicles presented a great challenge during formulation because of its tendency, as a hydrophilic drug, to escape from the nanovesicles to the external aqueous phase [74]. In the preliminary trials, freeze and thaw cycles were successful in increasing the EE% from 16 to 27% in TFH, while EE% was increased from 39 to 55% in MST. The effect of the consecutive freeze and thaw cycles on the EE% was previously studied by Costa et al. [75] where they found that the repetitive freeze and thaw cycles improved the liposomal EE% by reducing their lamellarity, forming more uniform dispersion and disrupting phospholipid bilayers, allowing more drug molecules to diffuse inside the nanovesicles. The EE% values observed with TFH ranged from 45.09 to 58.21%, whereas it ranged from ranged from 38.03 to 56.17% in MST. Statistical analysis of the EE% data shows that the entrapment of RM inside spanlastic vesicles was significantly affected by the Span type with *p* values of 0.0228 and 0.0002 in TFH and MST, respectively, as shown in Figs. 1d and 2d. The amount of RM encapsulated within the spanlastic vesicles formulated using Span^®^60 was higher than those formulated using Span^®^80. This may be explained based on the phase transition temperature (*T*_*c*_) of both surfactants which is higher in Span^®^60. The higher *T*_*c*_ maintains the hydrophobic structure of Span^®^60 in the completely extended and ordered orientation which may prevent RM leakage from the formulated spanlastic vesicles [76]. On the other hand, the lower EE% values observed with Span^®^80 may be attributed to the unsaturated alkyl chains in Span^®^80. The unsaturation of the alkyl chain may cause its tilting during preparation, which increases the vesicular membrane fluidity and subsequently its permeability, with higher chance for RM diffusion out of the prepared spanlastics [77].

### Optimization of the spanlastic variables

The independent formulation variables for the preparation of RM-loaded spanlastics are optimized in both preparation techniques aiming to minimize the PS and PDI of the formulated nanovesicles, while maximizing their ZP values and EE% as shown in Table 1. In TFH, Design-Expert 12^®^ software suggested “TFH 8” as the optimum system with desirability factor 0.758 which is prepared using Span^®^80 in ratio 4:1 with Brij 35 and sonicated for 1 min, whereas “MST 2” was chosen from those prepared using MST method with desirability factor of 0.773. MST 2 was formulated using Span^®^60 in a ratio 1:1 with Brij 35 and sonicated for 1 min. In order to ensure the validity of the built models in predicting the optimized formula, the two optimum systems are prepared and characterized where the observed and the predicted results for both systems are shown in Table 5. The two optimum systems, namely, TFH 8 and MST 2, were used for further characterization.

**Table 5:** The predicted and observed values of the optimized RM-loaded spanlastics prepared by TFH and MST methods

Method of preparation	Response	PS (nm)	PDI	ZP (mv)	EE (%)
TFH 8	Predicted values	141.425	0.296	(-) 45.420	52.317
	Observed values	183.015 ± 37.185	0.358 ± 0.092	(-) 45.60 ± 3.148	50.84 ± 1.9815
MST 2	Predicted values	94.426	0.608	(-) 37.079	54.691
	Observed values	84.060 ± 5.510	0.604 ± 0.046	(-) 39.30 ± 1.294	53.60 ± 1.9050

*PS* particle size, *PDI* polydispersity index, *ZP* zeta potential, *EE%* entrapment efficiency percentage, *TFH* thin film hydration, *MST* modified spraying technique

### Characterization of the optimized systems

#### In vitro RM release from the spanlastic vesicles

**Fig. 3:**
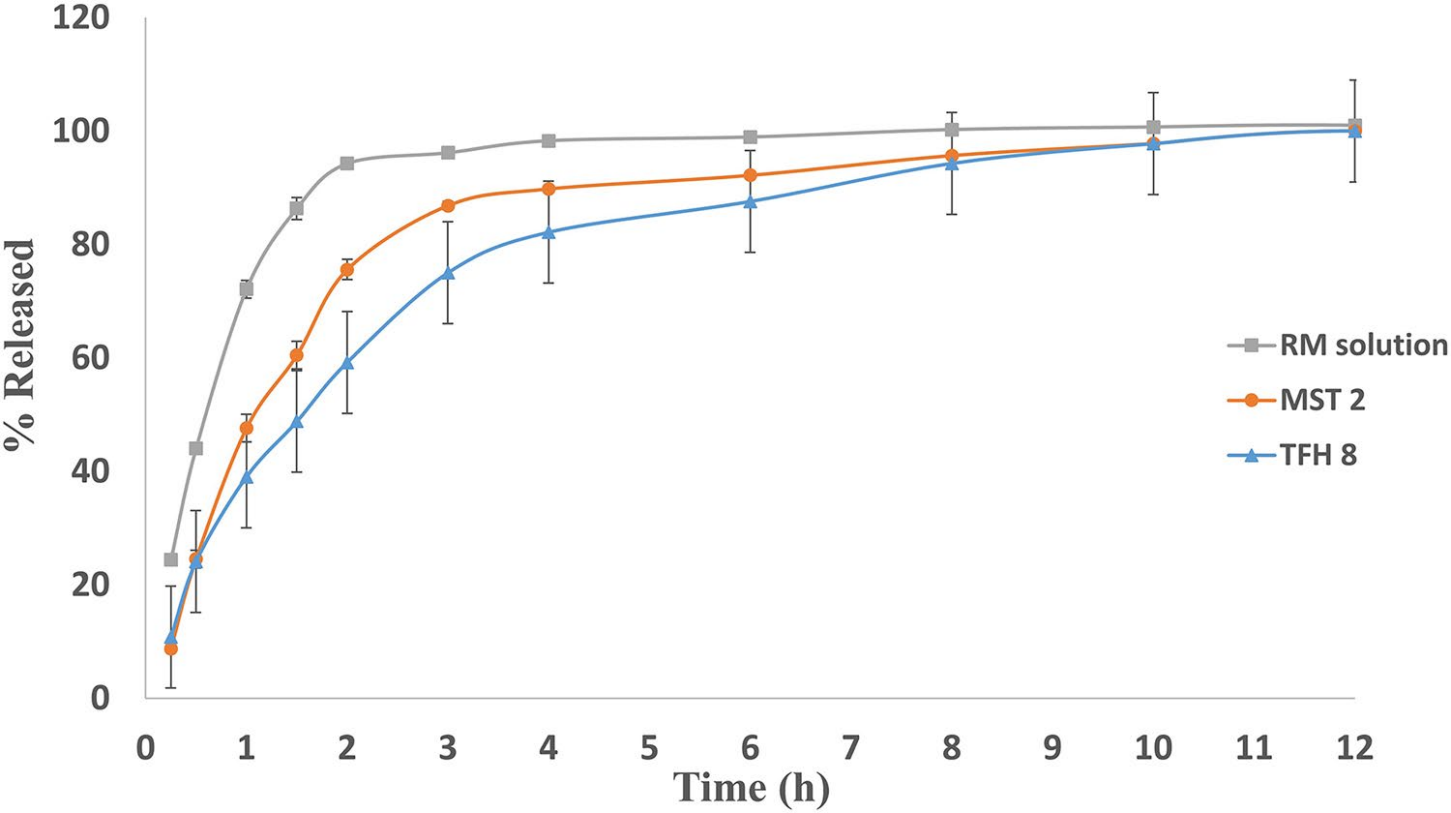
In vitro release profile of RM from TFH-optimized system (TFH 8), MST-optimized system (MST 2), and RM solution (RM concentrations are represented as mean ± SD)

RM release from RM solution and the optimum systems (TFH 8 and MST 2) are presented in Fig. 3. As observed, the complete release of RM from the two optimum systems was extended up to 10 h compared to 4 h in case of RM solution. An initial burst release can be observed from both formulations, which may be accounted for the unentrapped portions of RM available in the nanovesicular dispersion and surface drug molecules that can be readily released to the external compartment [45, 78]. In addition, the hydrophilic nature of RM may prevent its deep impediment inside the lipid bilayer and promote its tendency to escape from the formulated vesicles to the release media contributing to the initial burst release [79]. It is also obvious that RM release from MST 2 was more rapid compared to TFH 8. These observations coincide with the MDT values which were calculated to be 0.333 ± 0.0991, 1.365 ± 0.3118, and 2.115 ± 0.1073 h for RM solution, MST 2, and TFH 8, respectively. The faster RM release observed with MST 2 compared to TFH 8 can be explained in terms of the difference in their PS. The smaller vesicles of MST 2 show larger effective surface area with higher dissolution and release rate [80]. On the other hand, the difference in the vesicles composition due to the different Span: Brij 35 ratio could be another reason for the higher release rate observed with MST 2. Increasing Brij 35 concentration as an EA may result in an increased membrane permeability [81], where the high hydrophilicity of Brij 35 demonstrated in its high HLB value (16) [82] may produce an ultra-deformable vesicles containing transient pores in the vesicular membrane that could enhance drug leakage and result in faster drug release rate [83].

#### Transmission electron microscope (TEM)

**Fig. 4:**
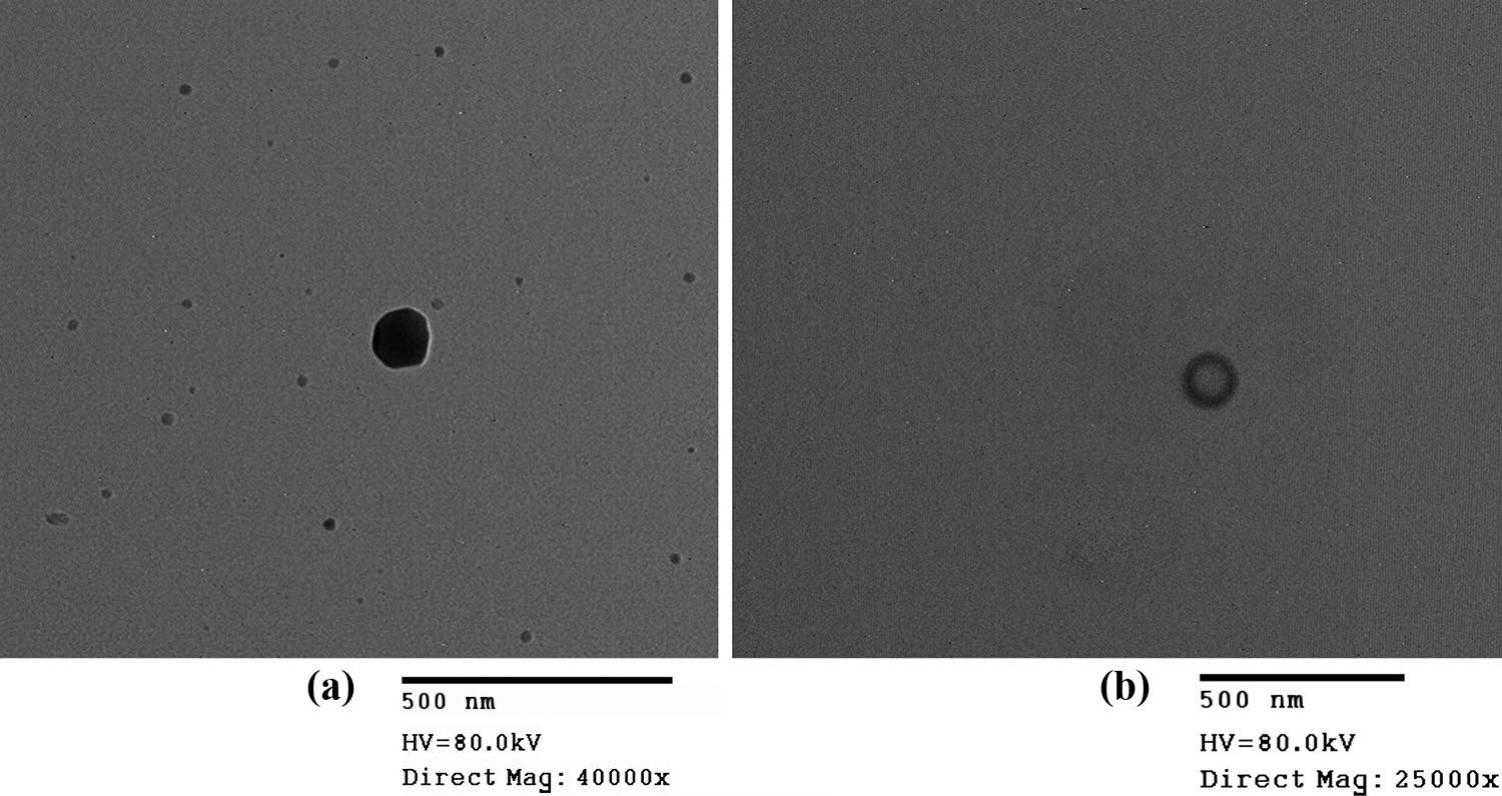
Transmission electron micrographs of TFH-optimized system (TFH 8) (**a:** magnification power = 40,000 ×) and MST-optimized system (MST 2) (**b:** magnification power = 25,000 ×)

The transmission electron micrographs of TFH 8 (a) and MST 2 (b) are represented in Fig. 4. The optimum RM-loaded spanlastic systems prepared by both techniques showed the non-aggregated, spherical nature of the formulated spanlastic nanovesicles. However, the optimized vesicles formulated via MST showed a characteristic distinct opaque shell surrounding hollow vesicles which could be accredited to the spraying technique used in their preparation [84].

#### pH measurement

The pH of the optimum systems was measured to evaluate their safety upon application onto the nasal mucous membrane. It was reported that the pH values of nasal preparations below 5.5 or higher than 6.5 may cause nasal irritation and affect the rate of drug penetration through the nasal epithelium especially when the applied formula has a higher buffer capacity [85]. In our work, the measured pH of the optimized systems were 6.378 ± 0.1706 and 6.289 ± 0.0613 for the TFH 8 and MST 2, respectively, which fall within the acceptable range for the intranasal preparations suggesting its safe, non-irritant effect on the nasal mucosa [86].

### In vivo biodistribution of RM

**Fig. 5:**
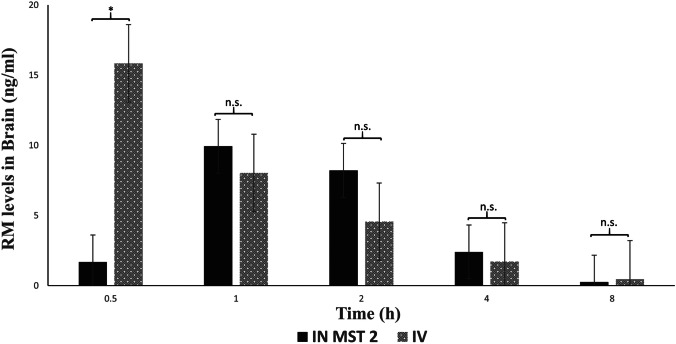
Mean RM concentration (mean ± SD) in brain upon IN administration of MST 2 and IV RM solution to healthy rats (*n* = 3). *significant difference and n.s, non-significant difference

MST 2 was chosen for the in vivo evaluation as it shows higher desirability factor [87] with lower PS and higher EE% when compared to TFH 8. Thus, MST 2 was administered intranasally at a dose of 0.05 mg/kg/day and compared to an intravenous RM solution. The concentrations of RM in plasma and brain tissues were measured by LC–MS/MS. The calibration curves of RM in plasma and brain homogenates were constructed in concentrations ranged from 0.1 to 1000 ng/mL with *R*^2^ values of 0.9996 and 0.9992, respectively. The pharmacokinetic parameters are calculated for the intranasal optimized system (IN-spanlastics) and the intravenous RM solution (IV-RM) to evaluate the brain-targeting behavior of MST 2 as presented in Table 6. The concentrations of RM inside brain at the different time intervals for both systems are shown in Fig. 5. It can be observed that the *T*_max_ was shorter for the IV-RM when compared to IN-spanlastics which may be accounted for the intranasal systemic absorption and the time required for the spanlastics to pass through the olfactory pathway. It can also be observed that the IV solution showed a higher *C*_max_; however, the AUC_0-∞_ of the intranasally administered MST 2 is insignificantly higher than the AUC_0-∞_ of IV solution (*p* = 0.422) as presented in Table 6. In order to ensure the brain-targeting behavior of RM spanlastics, BTE% and DTP% of MST 2 were calculated. The BTE% value was 458.472%, which demonstrates the wider distribution of the drug in the brain tissues relative to the systemic circulation and confirms the high targeting efficiency of the IN-spanlastics and its ability to cross the BBB. In addition, the positive value of DTP% (54.90%) indicates that the olfactory nerves were the main entry pathway to the brain [88]. These results could be attributed to the small PS of MST 2, which potentiated its ability to pass through the small pores of the olfactory mucosa and the tight junctions of BBB despite the possible repulsion between the negative nasal lining mucosa [89] and negatively charged spanlastics [63]. The same results were obtained and explained by Sahagun et al. [90] who concluded that the passage of nanoparticles across semipermeable membranes is more dependent on PS than surface charge. This is also supported by Fick᾽ law of diffusion which states that the passive diffusion of particles across semipermeable membrane is greatly dependent on their size and surface area [91]. Finally, the mean residence time (MRT), which indicates the average time that a molecule stays in the brain [92], was insignificantly higher in MST 2. These results suggest that the IN administration of the optimized RM-loaded spanlastics can achieve comparable effects to those obtained using IV drug solution and can be considered as alternative, non-invasive and efficient route for brain targeting. However, the high BTE% observed with the intranasally administered MST 2 signifies its possible superiority in reducing the undesirable systemic side effects observed with the CNS-acting drugs [93].

**Table 6:** Mean pharmacokinetic parameters of RM in brain following intranasal administration of RM spanlastics (IN MST 2) and intravenous RM solution (IV solution)

Pharmacokinetic parameter	IN MST 2	IV RM solution
*C*_max_ (ng/mL)	9.929 ± 0.40	15.835 ± 0.34
*T*_max_ (hr)	1	0.5
AUC_0-∞_ (ng.hr/mL)	28.840 ± 8.94	28.001 ± 1.84
*K* (hr^−1^)	0.570 ± 0.13	0.377 ± 0.13
*T*_1/2 half_ (hr)	1.200 ± 0.41	1.830 ± 0.24
MRT (hr)	2.420 ± 0.31	2.270 ± 0.71
BTE %	458.4720527	
DTP %	54.90261622	

*MST* modified spraying technique, *RM* rasagiline mesylate, *C*_*max*_ maximum concentration, *T*_*max*_ time to reach maximum concentration, *AUC* area under curve, *K* elimination rate constant, *MRT* mean residence time, *BTE%* brain-targeting efficiency percentage, *DTP%* direct transfer percentage

## Conclusion

In this study, two different preparation techniques, namely, MST and TFH, were utilized to formulate RM-loaded spanlastic vesicles. The investigated formulation variables affecting each technique were statistically characterized, compared, and optimized to prepare physically stable spanlastic vesicular system with small PS (< 100 nm) and high EE%. Accordingly, the optimized system prepared by MST showed smaller PS and higher EE%, so it was selected for further in vitro and in vivo evaluation. The in vivo pharmacokinetic results revealed that the extent of RM distribution to the brain was comparable for the intranasally administered MST 2 and the IV drug solution; however, the optimized spanlastic vesicular system has shown a significantly high BTE% which indicates the higher proportion of drug reaching the brain relative to the plasma after IN administration compared to the IV route. These results could be attributed to the small PS of the vesicles which permitted its direct passage through the olfactory pathway to the brain and suggested that the formulated vesicles could be a promising system for the efficient delivery of RM to brain tissues to exert its pharmacological activities without being dissipated to other body organs which subsequently may result in higher pharmacological efficiency and better safety profile. Formulation of an intranasal in situ gel containing RM-loaded spanlastics can be considered as a future prospect to enhance the retention of the drug in the nasal cavity and provide more sustainment for RM release for better patients᾽ compliance.

## Data Availability

The datasets generated during and/or analyzed during the current study are available from the corresponding author on reasonable request.

## Abbreviations

ANOVA: Analysis of variance
AUC: Area under curve
AUMC: Area under first moment curve
BBB: Blood-brain barrier
BTE%: Brain-targeting efficiency
CNS: Central nervous system
DTP%: Direct transfer percentage
EA: Edge activator
EE %: Entrapment efficiency percentage
HLB: Hydrophilic lipophilic balance
IN: Intranasal
IV: Intravenous
IS: Internal standard
LC–MS/MS: Liquid chromatography mass spectrometry
MAO-B: Monoamine oxidase type B
MDT: Mean dissolution time
MRT: Mean residence time
MST: Modified spraying technique
PD: Parkinson᾽s disease
PDI: Polydispersity index
PRESS values: Predicted residual error sum of squares value
PS: Particle size
RM: Rasagiline mesylate
*T*_*c*_: Transition temperature
TEM: Transmission electron microscopy
TFH: Thin film hydration
UV: Ultraviolet
ZP: Zeta potential
